# SARS-CoV-2 Detection in Gingival Crevicular Fluid

**DOI:** 10.1177/0022034520970536

**Published:** 2020-11-02

**Authors:** S. Gupta, R. Mohindra, P.K. Chauhan, V. Singla, K. Goyal, V. Sahni, R. Gaur, D.K. Verma, A. Ghosh, R.K. Soni, V. Suri, A. Bhalla, M.P. Singh

**Affiliations:** 1 Unit of Periodontics, Oral Health Sciences Centre, Post Graduate Institute of Medical Education and Research (PGIMER), Chandigarh, India; 2 Department of Internal Medicine, PGIMER, Chandigarh, India; 3 Department of Virology, PGIMER, Chandigarh, India; 4 Dr. Harvansh Singh Judge Institute of Dental Sciences & Hospital, Panjab University, Chandigarh, India

**Keywords:** COVID-19, oral health, oral hygiene, saliva, diagnostics, periodontal

## Abstract

Understanding the pathophysiology of the coronavirus disease 2019 (COVID-19) infection remains a significant challenge of our times. The gingival crevicular fluid being representative of systemic status and having a proven track record of detecting viruses and biomarkers forms a logical basis for evaluating the presence of severe acute respiratory syndrome coronavirus 2 (SARS-CoV-2). The study aimed to assess gingival crevicular fluid (GCF) for evidence of SARS-CoV-2 in 33 patients who were deemed to be COVID-19 positive upon nasopharyngeal sampling. An attempt was also made to comparatively evaluate it with saliva in terms of its sensitivity, as a diagnostic fluid for SARS-CoV-2. GCF and saliva samples were collected from 33 COVID-19–confirmed patients. Total RNA was extracted using NucliSENS easyMAG (bioMérieux) and eluted in the elution buffer. Envelope gene (*E* gene) of SARS-CoV-2 and human RNase P gene as internal control were detected in GCF samples by using the TRUPCR SARS-CoV-2 RT qPCR kit V-2.0 (I) in an Applied Biosystems 7500 real-time machine. A significant majority of both asymptomatic and mildly symptomatic patients exhibited the presence of the novel coronavirus in their GCF samples. Considering the presence of SARS-CoV-2 RNA in the nasopharyngeal swab sampling as gold standard, the sensitivity of GCF and saliva, respectively, was 63.64% (confidence interval [CI], 45.1% to 79.60%) and 64.52% (CI, 45.37% to 80.77%). GCF was found to be comparable to saliva in terms of its sensitivity to detect SARS-CoV-2. Saliva samples tested positive in 3 of the 12 patients whose GCF tested negative, and likewise GCF tested positive for 2 of the 11 patients whose saliva tested negative on real-time reverse transcription polymerase chain reaction. The results establish GCF as a possible mode of transmission of SARS-CoV-2, which is the first such report in the literature, and also provide the first quantifiable evidence pointing toward a link between the COVID-19 infection and oral health.

## Introduction

With the coronavirus disease 2019 (COVID-19) pandemic being firmly established, understanding the pathophysiology of this novel entity has been the challenge of our times. Being a never before encountered pathogen, the severe acute respiratory syndrome coronavirus 2 (SARS-CoV-2) has afforded no luxuries to those attempting to build knowledge in the face of this continuously mounting challenge. An essential part of improving understanding is to extrapolate what is already known. This has prompted the search for viral loads in various body fluids paralleling the presence of other viruses in these secretions. The search has entailed an assessment of viral titers in every fathomable body secretion such as cerebrospinal fluid (CSF), saliva, urine, feces, semen, breast milk, tears, and peritoneal fluid, of which samples of saliva, urine, feces, breast milk, and peritoneal fluid have demonstrated the presence of SARS-CoV-2 (Hung et al. 2003; Al Saiegh et al. 2020; Coccolini et al. 2020; Groß et al. 2020; Paoli et al. 2020; Sun et al. 2020; Xia et al. 2020). The evidence for most of these has so far been inconclusive. This, however, is true for most evidence pertaining to SARS-CoV-2, with most of the literature base being built upon a foundation of opinions, correspondence, and isolated clinical experiences. Valuable as these may be, there is a necessity to conduct proper clinical studies with standardized methodologies if we are to begin to draw some much-needed conclusions about how this virus behaves. A systematic review conducted on such meager data reveals olfactory and gustatory symptoms to be present in most patients with COVID-19, with a substantial majority of these even preceding general symptoms of the disease (Samaranayake et al. 2020).

The oral cavity has been touted as one of the most significant points of entry of the novel coronavirus; its location in confluence with the respiratory tract, as well as the drainage of saliva, predisposes it to be a major focus of interest as far as SARS-CoV-2 is concerned. Oral bacteria, periodontopathogens, and, in general, periodontal disease have been associated with respiratory conditions and adverse outcomes thereof, particularly acting in a synergistic mechanism along with viruses. There is now definitive evidence of the recovery of SARS-CoV-2 from saliva and nasopharyngeal swabs so much so that these form the basis of testing methodology. The utilization of saliva as an alternative to naso- and/or oropharynx swabs and blood sampling has been recommended based upon the clinical advantages of it being less invasive and more convenient for patients, particularly in terms of disease monitoring and cases where multiple testing is required (Sapkota et al. 2020). However, saliva is not the only fluid present in the oral cavity.

The gingival crevicular fluid (GCF) is a serum exudate that drains into the gingival sulcus after traversing the connective tissue and carries within it molecular and cellular components of importance such as antibodies, components of the complement system, plasma cells, and neutrophils. The collection and, in turn, analysis of GCF have long been a recognized and even popular approach to study conditions of the periodontium (Taylor et al. 2016). GCF has a proven track record of being amenable to the detection of viral shedding, with viruses such as the human cytomegalovirus and herpes simplex virus being recovered in sampling (Grenier et al. 2009).

With a background of this knowledge, it would only make sense to make an attempt in order to ascertain shedding of SARS-CoV-2 in the GCF. An attempt was also made to compare the sensitivity of GCF sampling results with those of saliva and nasopharyngeal swabs and do a comparative evaluation of results obtained with GCF sampling to those with saliva sampling in the same cohort of patients. It seems logical to make such an effort into the role GCF might play in the pathophysiology of COVID-19 and how it behaves as not only a possible mode of infection transfer but also a possible body fluid to be purposed for diagnostics. The established gold standard of nasopharyngeal swab (NPS) sampling, as advantageous as it may seem, is not without its drawbacks, the most glaring of which is the possibility of inducing a gag reflex (an aerosol-generating act) during sample collection, which poses a threat of infection transfer to health care professionals and others in the vicinity of sample collection. Some reports from over the world have mentioned the thin running supply of test swabs, which are a necessity for naso/oropharynx sampling.

Salivary samples that are obtained for COVID-19 testing are not purely representative of saliva as they further contain sputum and GCF, not to mention that saliva sample collection by means of spitting is itself an aerosol-generating act. Even the simple act of spitting into a container, as is undertaken in saliva sample collection, might possibly aerosolize the contagion and has led to recommendations of using straws during sample collection (Ceron et al. 2020). Also, such saliva collection methods are not suitable for patients who cannot expectorate saliva, such as patients who are unconscious. For these patients, suction aspiration of saliva is required (To et al. 2017). Moreover, a number of saliva sampling practices, even in self-collected samples from patients, mandate that this be done preferably in a condition wherein the patient has not had any food or drinks or has brushed his or her teeth after waking in the morning until the sample is collected, a somewhat cumbersome set of instructions to follow.

On the other hand, collection of a GCF sample is relatively noninvasive and repeatable. It does not require any special instructions to be followed by the patient and can be collected at the health care center immediately as and when the patient reports. In light of such obvious drawbacks to so-called established sampling practices, it seems sensible to explore GCF as a possible diagnostic fluid as well.

## Methods

### Patients

The study was carried out by the Unit of Periodontics, Oral Health Sciences Centre, in collaboration with the Department of Internal Medicine and Department of Virology, Postgraduate Institute of Medical Education and Research, Chandigarh, India. The study was performed after obtaining due approval from the Institute Ethics Committee (IEC no. NK/6404/Study/573). A total of 33 patients presenting to the Communicable Diseases Ward of the institution between July 1, 2020, and July 25, 2020, were recruited into the study after their COVID-19 status was confirmed by nasopharyngeal swab testing. Written informed consent was obtained. The sample size was based on convenience sampling owing to the fact that the study setting was a dedicated COVID center and the close proximity required on part of the health care worker with a potentially infectious patient to collect a GCF sample. However, as no sample size estimation was done a priori, a post hoc power analysis was performed to validate the same. Pregnant women and patients unwilling to give written informed consent were excluded from the study.

### Data Collection

Demographic data were recorded. Oral manifestations such as the presence or absence of ulcers, enanthem, and petechiae were also recorded using a mouth mirror.

### Sample Collection

GCF and saliva samples were collected from 33 COVID-19–confirmed patients by trained health care personnel by taking adequate protective measures as per the institute’s guidelines.

#### GCF

In total, 2 µL GCF was collected using color-coded 1- to 5-μL calibrated volumetric Hirschmann’s microcapillary pipettes. The sites were appropriately dried and isolated using cotton rolls to prevent salivary contamination, and the GCF was collected extracrevicularly by placing the micropipette gently at the entrance of the gingival sulcus, without inserting it into the crevice. Micropipettes were perceived to be better at sample collection in light of the inherent limitation of paper strips having nonspecific analytes attached to them (Pradeep et al. 2011). Even though such an analyte attachment has not yet been proven for paper strips as far as SARS-CoV-2 sampling is concerned, the fact that it is an established drawback while sampling for others led us to abstain from using this methodology. In patients with periodontitis, the sample was collected from the deepest periodontal pocket, whereas in healthy mouths, the sample was pooled from multiple sites until the required amount of 2 µL was obtained. One sample was discarded due to contamination with blood, and subsequent sampling was done from a similar pocket depth site at a different location in the mouth. The samples collected were transferred to 198 µL viral transport medium (HiViral HiMedia Laboratories) in sterile 0.5-mL microcentrifuge tubes.

#### Saliva

Saliva collection was done 2 to 3 h following GCF sample collection. The patients were asked to refrain from eating, drinking, brushing their teeth, or using mouth rinses at least 2 h prior to sample collection. Saliva samples were then collected by expectorating 0.5 to 1 mL of unstimulated whole saliva into sterile sputum containers and adding 2 mL of viral transport (Fakheran et al. 2020).

All the GCF and saliva samples were transported in a cold chain to the Department of Virology for further processing for the detection of SARS-CoV-2 RNA by real-time polymerase chain reaction (PCR). Finally, the RNA was eluted in the elution buffer (40 µL for GCF and 30 µL for saliva). There was loss of 2 saliva samples due to leakage during transportation.

### RNA Extraction

Total RNA was extracted from 200 µL of sample by using NucliSENS easyMAG (bioMérieux) according to the manufacturer’s instructions.

### Real-Time Reverse Transcriptase PCR

Envelope gene (*E* gene) of SARS-CoV-2 and human RNase P gene as internal control were detected in GCF and saliva samples by using the TRUPCR SARS-CoV-2 RT qPCR kit V-2.0 (I) in an Applied Biosystems 7500 real-time machine. Quality of reverse transcription PCR (RT-PCR) reaction was ensured by using appropriate controls. Distilled water was used as nontemplate control, while positive control, provided with the kit, RNase P was used as PCR reaction control for each set of experiments. Gene (internal control) was targeted as sample collection and extraction control.

### Statistical Analysis

Descriptive and inferential statistics were performed using IBM SPSS Statistics, version 23 (SPSS, Inc.). Data did not show a normal distribution (Kolmogorov-Smirnov test, Shapiro-Wilk test); thus, nonparametric tests were used in the present study. Inferential statistics such as the Mann-Whitney *U* test and Spearman correlation were used. The significance level was set at 0.05. Since no sample size calculation was undertaken a priori, a post hoc power analysis was performed using G* power, version 3.1 (HHU Dusseldorf). With an effect size of 1.25 between nasopharyngeal and GCF samples, given an α error of 5%, the post hoc power analysis revealed a power of 97.84%.

## Results

The demographic data are presented in detail in Table 1. Of the 33 patients included in the study, 19 (57.57%) were male and the rest were female. The mean age of the patient cohort was 43.96 y. Twenty-one of these 33 patients had no preexisting medical conditions. The 12 patients who reported to have medical histories included conditions such as diabetes (7), hypertension (7), epilepsy (1), hypothyroidism (1), and coronary artery disease (1). Three patients were morbidly obese. One out of the entire cohort of 33 patients presented with a dermatological finding, which included rashes on both legs.

**Table 1.** Demographic and Disease Course Data.

COVID-19 Status: Symptomatic/Asymptomatic							Ct Value of *E* Gene		
	Age, y	Sex	Underlying Systemic Condition	Gum Disease	Oral Findings	Day of Illness	Nasopharyngeal Swab	GCF	Saliva
Symptomatic	64	Male	Diabetes		Ageusia	2 d	18.4	27.42	22.63
	27	Male			Caries	2 d	17.7	23.7	17.23
	23	Male		Present	Gum bleed, caries	2 d	14.1	28.69	ND
	40	Male			Perioral swelling	2 d	17.5	27.39	23.16
	64	Male		Present	Gingival erythema, caries	2 d	18.5	28.64	26.6
	52	Male	Diabetes, hypertension	Present	Gingival erythema	3 d	17.2	27.25	26.32
	65	Female	Hypertension		—	3 d	17	24.85	27.39
	28	Male		Present	Gingival erythema	4 d	22.9	27.62	IS
	25	Female		Present	Gum bleed	4 d	24	ND	33.5
	68	Male	Diabetes, hypertension, CAD	Present	Recession, caries	4 d	26.8	23.24	IS
	29	Male			Perioral swelling	4 d	27	ND	ND
	34	Female	Epilepsy		—	4 d	24.4	36.71	26.71
	31	Female			—	5 d	34.8	36.85	33.92
Asymptomatic	33	Female		Present	Gum bleed, gingival erythema	—	12.1	23.17	25.29
	52	Female			—	—	21.8	28.52	31.35
	34	Male			—	—	31.3	27.48	35.02
	33	Male			—	—	33.08	ND	ND
	54	Female			—	—	29.35	ND	37.06
	28	Female			—	—	28	ND	ND
	51	Female	Hypertension	Present	—	—	16.7	ND	38.34
	57	Male	Diabetes		—	—	26.9	26.87	31.43
	53	Male	Hypertension		—	—	25.6	29.41	28.8
	25	Male		Present	Gum bleed, gingival erythema	—	19	21.53	24.62
	56	Male		Present	Petechiae, gum bleed, gingival erythema	—	17	24.57	ND
	38	Male			—	—	22.15	25.34	25.05
	62	Male	Diabetes, Hypertension	Present	Gingival erythema, caries, perioral swelling	—	33.2	ND	ND
	59	Male	Diabetes		—	—	33	ND	ND
	21	Male			—	—	22.06	29.14	28.7
	57	Female	Morbidly obese		—	—	31.6	ND	ND
	48	Female	Morbidly obese	Present	Gingival erythema	—	24.7	ND	ND
	45	Female	Hypothyroidism, obese	Present	Gum bleed, caries	—	26.1	ND	ND
	35	Female			—	—	28.9	ND	ND
	56	Female	Diabetes, hypertension	Present	Gum bleed, perioral swelling	—	14.6	23.16	22.49

CAD, coronary artery disease; COVID-19, coronavirus disease 2019; Ct value, cycle threshold value; GCF, gingival crevicular fluid; IS, insufficient sample; ND, not detected.

As far as COVID-19 status is concerned, 20 of these 33 patients were asymptomatic carriers (60.60%) and 13 presented with mild symptoms of fever, cough, and/or sore throat (39.4%). Fourteen of these 33 patients (42.42%) were deemed to have gum disease upon examination.

On examination, 17 patients presented with oral findings (51.51%), which included ageusia (1), petechiae (1), gum/gingival recession (1), gingival erythema (8), dental caries (6), perioral swelling (4), and gum bleed (7), of which only ageusia, petechiae, and perioral swelling were reported to be associated with COVID-19–infected patients.

We did not find any significant association between the presence of periodontal disease or oral findings and SARS-CoV-2 detection in GCF. Five cases of periodontal disease and 7 of the periodontally healthy group tested negative in GCF. Similarly, 5 cases with oral findings and 7 cases with no oral findings tested negative for GCF. Also, no significant correlation was observed between negative GCF sampling and a lack of symptoms. Six patients presenting symptoms of COVID-19 and 7 asymptomatic cases tested negative for GCF.

### Detection of SARS-CoV-2 RNA in GCF Samples

*E* genes of SARS-CoV-2 were detected in 63.64% (*n* = 21/33; confidence interval [CI], 45.12% to 79.60%) of GCF samples and 64.52% (*n* = 20/31; CI, 45.37% to 80.77%) of saliva samples.

Saliva samples tested positive in 3 of the 12 patients whose GCF tested negative, and likewise, GCF tested positive for 2 of the 11 patients whose saliva tested negative on real-time reverse transcription polymerase chain reaction (rRT-PCR). As compared to cycle threshold (Ct) values of nasopharyngeal swabs (*E* gene, 23.55 ± 6.31), both GCF and saliva samples were found to have a higher Ct value (GCF: *E* gene, 27.21 ± 3.91; saliva: *E* gene, 28.28 ± 5.42) (Fig.). There was a statistically significant difference of 6 to 9 mean Ct values change in GCF and saliva compared to nasopharyngeal swabs (Table 2).

**Figure.**
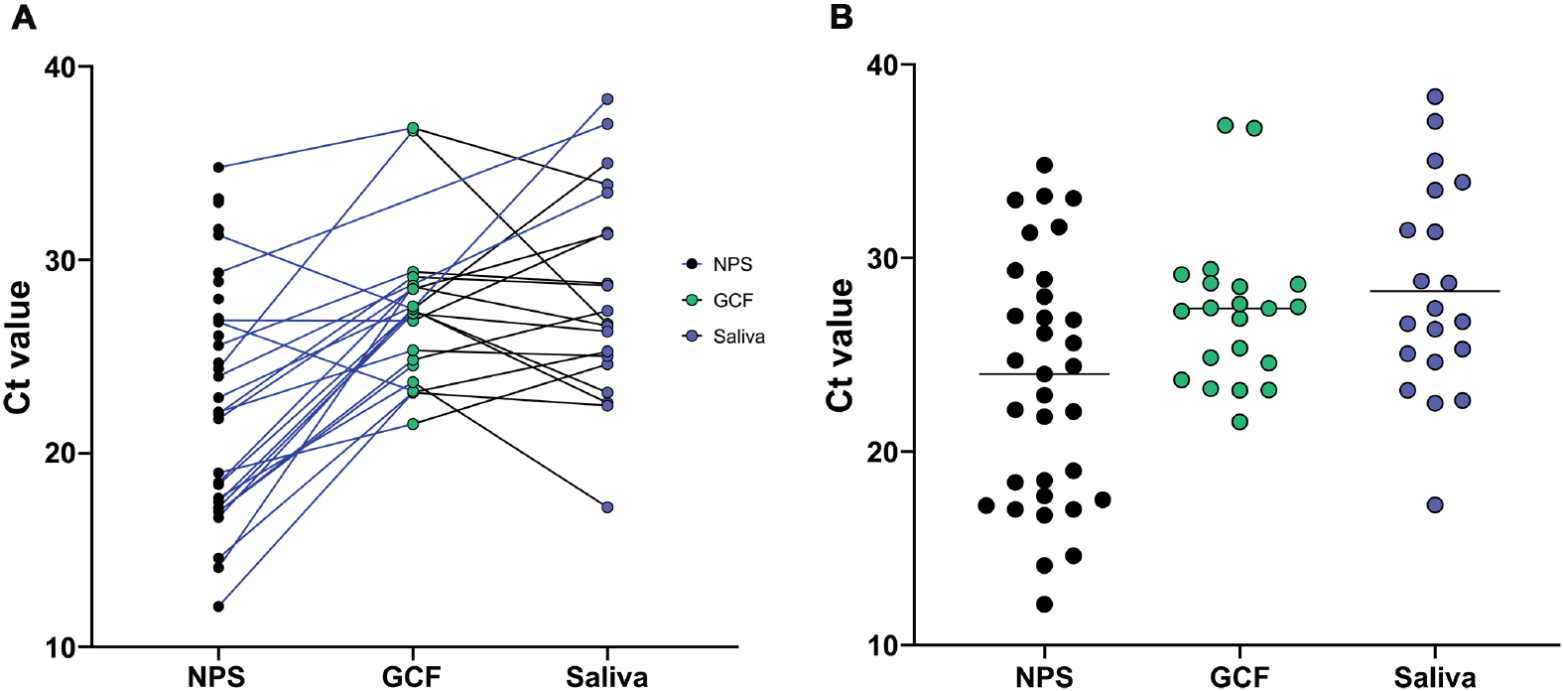
Comparison of Ct values between different clinical samples. (**A**) Patient matched samples, represented by the connecting lines. A blue line represents nasopharyngeal swab (NPS) pairing with gingival crevicular fluid (GCF) or saliva sample while a black line represents pairing between GCF and saliva. (**B**) All positive nasopharyngeal swabs (*n* = 33), GCF (*n* = 21), and saliva samples (*n* = 20) were compared. Mean Ct value is represented by a horizontal line in each group.

**Table 2.** Comparison and Correlation of Ct Values between the Different Samples.

Sample	*N*	Range of Ct Values of *E* Gene	Median	Mean ± Standard Deviation	*P* Value	Correlation Coefficient
NPS	21	12.10–34.80	19	21.03 ± 5.73	0.001^a^	0.441 (moderate positive linear relationship; *P* 0.045^a^)
GCF	21	21.53–36.85	27.39	27.21 ± 3.91		
GCF	18	12.00–36.85	27.32	26.63 ± 5.46	0.658	0.674 (moderate positive linear relationship; *P* 0.002^a^)
Saliva	18	13.00–35.02	26.46	26.09 ± 5.42		
NPS	20	12.10–34.80	20.4	21.55 ± 5.84	0.001^a^	0.561 (moderate positive linear relationship; *P* 0.010^a^)
Saliva	20	17.23–38.34	27.05	28.28 ± 5.42		

Ct value, cycle threshold value; GCF, gingival crevicular fluid; *N*, number of positive samples common to both groups; NPS, nasopharyngeal swab.

a Statistically significant.

Although the study was a cross-sectional study, an attempt was made to correlate the Ct values to the day of illness. The Ct values were found to increase linearly as the day of illness progressed from day 2 to day 5 (Table 1), indicating a reduction in viral shedding. This may be the reason why SARS-CoV-2 could not be detected from 2 samples of GCF and 1 of saliva collected on day 4.

## Discussion

Considering the presence of SARS-CoV-2 RNA in the NPS swabs as gold standard, the sensitivity of GCF and saliva, respectively, was found to be 63.64% (CI, 45.1% to 79.60%) and 64.52% (CI, 45.37% to 80.77%).

Periodontal health has been known to be reflective of systemic health. By this extension, GCF has been used in a number of studies to gauge the systemic status of individuals in terms of being indicative of the serum level of immune response. GCF sampling has also been used to reliably determine viral loads while studying periodontal conditions (Grenier et al. 2009). Armed with this knowledge, studying the GCF for the possibility of estimating the viral load of SARS-CoV-2 would seem only logical. Not only this, but GCF sampling has also been used to be reliably deterministic of the serum immune response and could further be extrapolated to be reflective of cytokine levels that seem manifest in COVID-19 cases (Sahni and Gupta 2020).

A significantly justifiable yet unanswered question seems to be why certain patients have more severe consequences of COVID-19 compared to others. While age, sex, and the presence of comorbidities do explain a number of these cases, a significant proportion of the population seems to comprise relatively young, healthy patients who do not fall into any of these traditional groups yet have adverse outcomes (Shi et al. 2020). It is certainly interesting to note that 5 of the 13 symptomatic patients had systemic compromise in the form of one of such comorbid conditions, while 6 of these 13 were periodontally compromised.

Such relations pertaining to the particular niche of the periodontal pocket acting as a reservoir for SARS-CoV-2 by replicating and further migrating to mix with the saliva and even entering systemic circulation have certainly been hypothesized (Badran et al. 2020).

GCF being exudative in nature, it would only make sense to state that if the oral hygiene of patients remains poor, it predilects one to have a greater amount of inflammatory exudate. This, in light of the fact that SARS-CoV-2 has been recovered from the GCF of patients, would lead one to postulate that poor oral hygiene could possibly increase the viral load in GCF. With the virus being recovered in GCF, it forms a further aspect of infectivity pertaining to SARS-CoV-2. Advocating maintenance of oral hygiene, hence, seems to be prudent advice.

The host ACE2 receptor plays a crucial role in establishing the infectivity of SARS-CoV-2. It has been expressed in the epithelium of the oral cavity, particularly in that of the oral tongue, buccal mucosa, and gingival tissues (Xu et al. 2020). It can be argued that the expression of the ACE2 receptor in the gingival epithelium and viral recovery in the GCF could form a basis for understanding a potential route of infection exhibited at this level and how the inflammatory status of the periodontium, which is essentially determined by oral hygiene, might influence the COVID-19 infection. Other probable mechanistic links have been presented in detail in the Appendix. The present study, however, did not seem to find a direct link between the recovery of SARS-CoV-2 in GCF and the presence/absence of gum disease. Probably greater sample sizes would be required to conclusively report on this association.

The results of the study also suggested that SARS-CoV-2 was recovered from both asymptomatic carriers and those who were mildly symptomatic. This seems to point toward a concerning subject wherein a number of dental practices are looking to open up across the world. The fact that there is a fluid in the gingival crevice that harbors SARS-CoV-2, even in asymptomatic individuals, is a troubling thought as this could potentially infect unwary health care professionals. In light of this, the results of the present study become important not only for practitioners but in framing of policy and screening measures to be instituted as dentistry begins to open up. It would be a severe blow to the credibility of our profession if, due to a lack of evidence, we were to somehow unknowingly contribute to a blunting of the response to the pandemic. Aerosol-generating procedure or not, the GCF would be involved in conceivably every procedure in the vicinity of the gingival sulcus, which for all practical purposes covers the entire purview of dentistry. By demonstrating recovery of SARS-CoV-2 in the GCF, this study establishes that this fluid contributes to the viral load being recovered in saliva samples. This would further call into question as to just how infective is saliva alone?

The perceived limitations of the study are that a larger sample size would be required to comment more definitively upon the oral hygiene status of an individual and how it relates to the presentation of the COVID-19 infection. Also, as the study design was cross-sectional, temporal associations could not be evaluated.

A future line of investigation could follow the “cytokine storm” profile of COVID-19 and as it reflects in the GCF and how it correlates in patients with the presence or absence of gum disease.

## Conclusion

GCF and saliva seem to be comparable in terms of their sensitivity to detect SARS-CoV-2. The comparability of GCF and saliva in terms of their sensitivity, as well as the advantages that GCF has over saliva sampling in certain cases, suggests that GCF could very well be purposed for diagnostics. It would not be unreasonable to state that procedures such as ultrasonic scaling or any procedure performed without a rubber dam would expose the dental health care provider to GCF (as it does to saliva) and pose a risk of infection transfer. In light of this knowledge, the demonstration of SARS-CoV-2 in GCF is a significant finding that goes a long way in understanding the COVID-19 infection and how it relates to oral health and the practice of dentistry.

## Author Contributions

S. Gupta, contributed to conception, design, data acquisition, analysis, and interpretation, drafted the manuscript; R. Mohindra, contributed to conception and data acquisition, critically revised the manuscript; P.K. Chauhan, contributed to data analysis and interpretation, drafted the manuscript; V. Singla, A. Ghosh, R.K. Soni, V. Suri, A. Bhalla, contributed to data acquisition, critically revised the manuscript; K. Goyal, R. Gaur, D.K. Verma, contributed to data analysis, and interpretation, critically revised the manuscript; V. Sahni, contributed to conception, data analysis, and interpretation, drafted the manuscript; M.P. Singh, contributed to conception, design, data analysis, and interpretation, critically revised the manuscript. All authors gave final approval and agree to be accountable for all aspects of the work.

## Supplemental Material

DS_10.1177_0022034520970536 – Supplemental material for SARS-CoV-2 Detection in Gingival Crevicular Fluid Click here for additional data file. Supplemental material, DS_10.1177_0022034520970536 for SARS-CoV-2 Detection in Gingival Crevicular Fluid by S. Gupta, R. Mohindra, P.K. Chauhan, V. Singla, K. Goyal, V. Sahni, R. Gaur, D.K. Verma, A. Ghosh, R.K. Soni, V. Suri, A. Bhalla and M.P. Singh in Journal of Dental Research
