# Supplementary material for: SARS-CoV-2 Detection in Gingival Crevicular Fluid
Source: J Dent Res. 2020 Nov 2;100(2):187–93. doi: 10.1177/0022034520970536 (PMC7642823; doi:10.1177/0022034520970536)
Supplement: DS_10.1177_0022034520970536 – Supplemental material for SARS-CoV-2 Detection in Gingival Crevicular Fluid [file DS_10.1177_0022034520970536.pdf]

Appendix

Title: SARS CoV-2 detection in Gingival Crevicular Fluid

Authors: Dr. Shipra Gupta, Dr. Ritin Mohindra, Dr. Poonam Kanta Chauhan, Dr. Vidhi Singla, Dr. Kapil Goyal, Dr. Vaibhav Sahni , Dr. Ritu Gaur, Dr. Dipesh Kumar Verma, Dr. Arnab Ghosh, Mr. Roop Kishor Soni, Dr. Vikas Suri, Dr. Ashish Bhalla, Dr. Mini P. Singh

Discussion

Probable mechanistic links between SARS CoV-2 & Oral Health

Oral bacteria, with their natural ease of access to the respiratory tract, can result in supra-added and aggravated infections secondary to COVID-19, which has formed a basis for advocating a link between oral hygiene and the severity of COVID-19 (Sampson et al. 2020). GCF, being representative of oral hygiene, viral load and immune response both at the local level of the gingival crevice and systemic level of serum, then becomes an obvious fluid to assess for SARS CoV-2 and how it relates to not only advocating a link between oral hygiene and infection severity but also to determine just how far reaching is the SARS CoV-2 in its infectivity and shedding.

It is also well established that a combined viral-bacterial etiology is linked to a number of systemic conditions by virtue of the synergistic association between viruses and periodontopathogens particularly those relating to respiratory disease (Slots 2010). There has been demonstration of Human cytomegalovirus (HCMV) and Epstein-Barr virus (EBV) DNA copies in periodontal pockets and gingival tissues. Not only this, but elevated herpes virus counts have paralleled the increase in severity of periodontitis. Saygun et al. (2005) found significant positive correlations amongst salivary and gingival tissue levels of HCMV and EBV, periodontal pocket depths and salivary EBV and HCMV DNA counts.

So much so, that it has been suggested that determination of herpes virus DNA in periodontal sites via RT-PCR may become a viable marker to monitor periodontal disease course (Kubar et al. 2005). Herpes simplex virus, Cytomegalovirus and periodontopathogens have been linked

to cardiovascular disease and diabetes as well, further establishing their role in systemic pathophysiology (Paquette et al. 2007; Mealey et al. 2007).

The periodontal status of an individual and its effect on the pathogenesis of respiratory infection can be explained through a variety of mechanisms such as the alteration of respiratory epithelium by cytokines elaborated as a result of periodontal conditions which promote further infection by respiratory pathogens. Enzymes related to periodontal disease can modify mucosal surfaces making them more amenable to adhesion and colonisation by respiratory pathogens. Such enzymes can also cause the destruction of bacterial salivary pellicles thereby impeding their clearance from mucosal surfaces. Apart from these more or less indirect mechanisms, a more direct one involving aspiration of oral or periodontal pathogens into the lungs may contribute to respiratory illness (Sampson et al. 2020).

Although being a viral disease itself, severe forms of COVID-19 infections have been observed to have bacteria play a role in determining adverse outcomes relating to pneumonia, sepsis, acute respiratory distress syndrome, shock (septic) and death. The comorbidities of cardiovascular disease, hypertension and diabetes have been associated with increased risk of COVID-19 related adverse outcomes. Incidentally, patients with these afflictions have been demonstrated to be suffering from periodontal disease and possessing altered oral biofilms. Periodontopathogens have been associated with pneumonia, bacteraemia and systemic inflammation (Sampson et al. 2020). It is certainly interesting to note that five of the thirteen symptomatic patients had systemic compromise in the form of one of such cormorbid conditions, while six out of these thirteen were periodontally compromised.

However, it is not only at this level where a commonality between the systemic aspect of COVID-19 infections and periodontal disease can be drawn (Gupta and Sahni 2020). The symptomatic presentation of COVID-19 seems to reflect a 'cytokine storm' at the systemic level with elevated serum levels of IL-10, IL-17, IL-1 beta, IL-2, IL-9, Il-8, G-CSF, GM-CSF, TNF alpha, IFN-gamma, MCP1, IP10, MIP1A and MIP1B (Shi et al. 2020). These along with an elevated response of the Th17 pathway have also been observed in patients of periodontal disease. (Sahni and Gupta, 2020)

COVID-19 infections have also been associated with the induction of a coagulopathy giving rise to a hypercoagulable state with its entailing disorders which include not only thrombosis but even vascular events of a fatal nature. This mechanism is potentially explainable as a result of a cytokine storm, Renin-Angiotensin System dysregulation and endothelial injury relating

to uncontrolled inflammation (Shi et al. 2020). Such relations pertaining to the particular niche of the periodontal pocket acting as a reservoir for the SARS CoV-2 by replicating and further migrating to mix with the saliva and even entering systemic circulation have certainly been hypothesised (Badran et al. 2020).

GCF being exudative in nature, it would only make sense to state that if the oral hygiene of patients remains poor it predilects one to have a greater amount of inflammatory exudate. This in light of the fact that SARS CoV-2 has been recovered from the GCF of patients would lead one to postulate that poor oral hygiene could possibly increase the viral load in GCF. The viral copies in GCF might be more than those in saliva. This may be justified when we take into account that mere 2µl of GCF is demonstrating CT values similar to 200 µl of saliva. With the virus being recovered in GCF, it forms a further aspect of infectivity pertaining to the SARS CoV-2. Advocating maintenance of oral hygiene, hence seems to be prudent advice.

The host ACE2 receptor plays a crucial role in establishing the infectivity of the SARS CoV-2. These have been found to be expressed in the epithelium of the oral cavity particularly in that of the oral tongue, buccal mucosa and gingival tissues (Xu et al. 2020). It can be argued that the expression of the ACE2 receptor in the gingival epithelium and its recovery in the GCF could form a basis for understanding a potential route of infection exhibited at this level and how the inflammatory status of the periodontium, which is essentially determined by oral hygiene, might influence the COVID-19 infection. The present study, however, did not seem to find a direct link between the recovery of the SARS CoV-2 in GCF and the presence/absence of gum disease. Probably greater sample sizes would be required in order to conclusively report on this association.

There is evidence in literature to suggest the involvement of enzymes such as furin and cathepsin-L in the cleavage of S (spike) protein of the SARS CoV-2 to facilitate its binding and subsequent entry into host cells via the ACE2 receptors. Chronic periodontitis can lead to the activation of these enzymatic pathways which could possibly enhance the virulence and subsequent infectivity of the SARS CoV-2 (Balaji et al. 2020). The lack of furin in basal cells of the host epithelium could be compensated by furin-like peptidases derived from the microbiota of the oral cavity. There is evidence in literature of viral entry being enhanced by such mechanisms involving *Streptococcus* (especially *S. gordonii V2016*) and Human Papilloma Virus (HPV) (Pavlova et al. 2018).

S. gordonii is quite commonplace in isolates obtained from peri-apical lesions of patients suffering from apical periodontitis and hence, with its furin-like activity could facilitate SARS CoV-2 binding and entry into host oral epithelial cells (Kim et al. 2017).

References

Balaji TM, Varadarajan S, Rao USV, Raj AT, Patil S, Arakeri G, Brennan PA. 2020. Oral cancer and periodontal disease increase the risk of COVID 19? A mechanism mediated through furin and cathepsin overexpression. Med Hypotheses. 144: 109936. doi: 10.1016/j.mehy.2020.109936

Gupta S, Sahni V. 2020. The intriguing commonality of NETosis between COVID-19 & Periodontal disease. Med Hypotheses. 144:109968. doi: 10.1016/j.mehy.2020.109968.

Kim AR, Ahn KB, Kim HY, Seo HS, Kum KY, Yun CH, Han SH. 2017. Streptococcus gordonii lipoproteins induce IL-8 in human periodontal ligament cells. Mol Immunol. 91: 218-224.

Kubar A, Saygun I, Özdemir A, Yapar M, Slots J. 2005. Real-time polymerase chain reaction quantification of human cytomegalovirus and Epstein-Barr virus in periodontal pockets and the adjacent gingiva of periodontitis lesions. J Periodontal Res. 40(2):97-104.

Mealey BL, Ocampo GL. 2007. Diabetes mellitus and periodontal disease. Periodontol 2000. 44(1):127-53.

Pavlova SI, Wilkening RV, Federle MJ, Lu Y, Schwartz J, Tao L. 2019. Streptococcus endopeptidases promote HPV infection in vitro. Microbiology open. 8(1): e00628.

Paquette DW, Brodala N, Nichols TC. 2007. Cardiovascular disease, inflammation, and periodontal infection. Periodontol 2000. 44(1):113-26.

Sampson V, Kamona N, Sampson A. 2020. Could there be a link between oral hygiene and the severity of SARS-CoV-2 infections?. Br Dent J. 228(12): 971–975.

Sampson V. 2020. Oral hygiene risk factor. Br Dent J. 228(8): 569.

Saygun I, Kubar A, Özdemir A, Slots J. 2005. Periodontitis lesions are a source of salivary cytomegalovirus and Epstein–Barr virus. J Periodon Res. 40(2):187-91.

Shi W, Lv J, Lin L. 2020. Coagulopathy in COVID-19: Focus on vascular thrombotic events. J Mol Cell Cardiol. 146:32-40.

Slots J. 2010. Herpesviral–bacterial interactions in periodontal diseases. Periodontol 2000. 52(1):117.
